# Community Transmission of SARS-CoV-2 at Two Family Gatherings — Chicago, Illinois, February–March 2020

**DOI:** 10.15585/mmwr.mm6915e1

**Published:** 2020-04-17

**Authors:** Isaac Ghinai, Susan Woods, Kathleen A. Ritger, Tristan D. McPherson, Stephanie R. Black, Laura Sparrow, Marielle J. Fricchione, Janna L. Kerins, Massimo Pacilli, Peter S. Ruestow, M. Allison Arwady, Suzanne F. Beavers, Daniel C. Payne, Hannah L. Kirking, Jennifer E. Layden

**Affiliations:** ^1^Chicago Department of Public Health; ^2^Epidemic Intelligence Service, CDC; ^3^Center for Surveillance, Epidemiology, and Laboratory Services, CDC; ^4^COVID-19 Response Team, CDC.

SARS-CoV-2, the virus that causes coronavirus disease 2019 (COVID-19), has spread rapidly around the world since it was first recognized in late 2019. Most early reports of person-to-person SARS-CoV-2 transmission have been among household contacts, where the secondary attack rate has been estimated to exceed 10% (*1*), in health care facilities (*2*), and in congregate settings (*3*). However, widespread community transmission, as is currently being observed in the United States, requires more expansive transmission events between nonhousehold contacts. In February and March 2020, the Chicago Department of Public Health (CDPH) investigated a large, multifamily cluster of COVID-19. Patients with confirmed COVID-19 and their close contacts were interviewed to better understand nonhousehold, community transmission of SARS-CoV-2. This report describes the cluster of 16 cases of confirmed or probable COVID-19, including three deaths, likely resulting from transmission of SARS-CoV-2 at two family gatherings (a funeral and a birthday party). These data support current CDC social distancing recommendations intended to reduce SARS-CoV-2 transmission. U.S residents should follow stay-at-home orders when required by state or local authorities.

During January 1–March 20, 2020, specimens that tested positive for SARS-CoV-2 at hospital, commercial, or public health laboratories were reported to CDPH; each triggered an epidemiologic investigation. Contact tracing interviews were conducted with patients with confirmed COVID-19 using a structured questionnaire designed to identify the date of symptom onset and any person with whom the patient had close contact since that date. The type of contact and setting in which the contact occurred were recorded. Close contacts of patients with confirmed or probable COVID-19 were interviewed and enrolled in active symptom monitoring using Research Electronic Data Capture software (REDCap, version 8.8.0, Vanderbilt University, 2020). Patients were classified as having confirmed COVID-19 if SARS-CoV-2 was detected by real-time reverse transcription–polymerase chain reaction testing of a nasopharyngeal or oropharyngeal specimen. Patients were classified as having probable COVID-19 if they developed new symptoms of fever, cough, or shortness of breath within 14 days of contact with a patient with confirmed or probable COVID-19 but did not undergo laboratory testing (consistent with CDC recommendations,* the Illinois Department of Public Health prioritizes testing for hospitalized patients and other high-risk groups).

In February 2020, a funeral was held for a decedent with a non-COVID-19, nonrespiratory cause of death. A close friend of the bereaved family (patient A1.1) attended the funeral; patients in this investigation were referred to by their family cluster letter (A or B), then by the assumed transmission generation (1–4), and finally, in sequence order within each generation (1–7)^†^ (Figure 1). Patient A1.1 had recently traveled out of state and was experiencing mild respiratory symptoms; he was only tested later as part of the epidemiologic investigation and received a diagnosis of confirmed COVID-19. The evening before the funeral (investigation day 1), patient A1.1 shared a takeout meal, eaten from common serving dishes, with two family members of the decedent (patients B2.1 and B2.2) at their home. At the meal, which lasted approximately 3 hours, and the funeral, which lasted about 2 hours and involved a shared “potluck-style” meal, patient A1.1 also reported embracing family members of the decedent, including patients B2.1, B2.2, B2.3, and B3.1, to express condolences.

**FIGURE 1:**
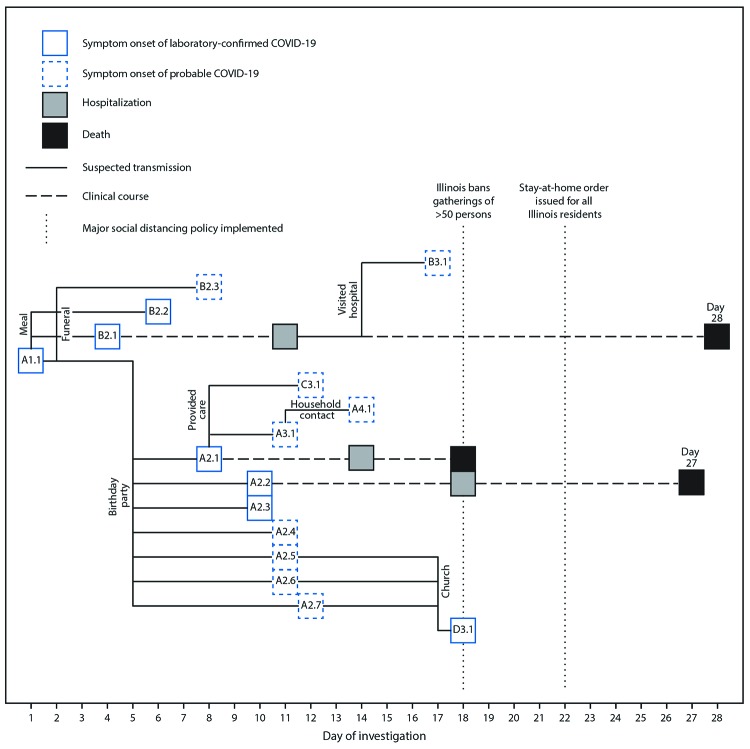
Timeline of events and symptom onsets, by day of investigation, in a cluster of COVID-19 likely transmitted at two family gatherings — Chicago, Illinois, February–March 2020 **Abbreviation: **COVID-19 = coronavirus disease 2019. **Notes: **Patients were designated by their family cluster letter (A or B), then by the assumed transmission generation (1–4), and finally, by sequence within each generation (1–7). Patient A2.1 died on investigation day 18; patient A2.2 died on investigation day 27; and patient B2.1 died on investigation day 28.

Patients B2.1 and B2.2 subsequently developed confirmed COVID-19 with onset of symptoms 2 and 4 days, respectively, after the funeral; patient B2.3 developed probable COVID-19 with symptom onset 6 days after the funeral (investigation day 8). Patient B2.1 was hospitalized on investigation day 11, required endotracheal intubation and mechanical ventilation for acute repiratory failure, and died on investigation day 28. Patients B2.2 and B2.3 were managed as outpatients, and both recovered.

During investigation days 11–14, another family member who had close physical contact with patient A1.1 at the funeral (patient B3.1) visited patient B2.1 on the acute medical inpatient ward, embraced patient B2.1, and provided limited personal care, while wearing no personal protective equipment (PPE). Patient B3.1 developed signs and symptoms consistent with COVID-19, including a fever and cough on investigation day 17, 3 days after last visiting B2.1. Patient B3.1 had also attended the funeral 15 days before symptom onset but described more extensive exposure while visiting patient B2.1 in the hospital.

Three days after the funeral, on investigation day 5, patient A1.1, who was still experiencing mild respiratory symptoms, attended a birthday party attended by nine other family members, hosted in the home of patient A2.1. Close contact between patient A1.1 and all other attendees occurred; patient A1.1 embraced others and shared food at the 3-hour party. Seven party attendees subsequently developed COVID-19 3–7 days after the event (Figure 2), including three with confirmed cases (patients A2.1, A2.2, and A2.3) and four with probable cases (patients A2.4, A2.5, A2.6, and A2.7). Two patients with confirmed COVID-19 (A2.1 and A2.2) were hospitalized; both required endotracheal intubation and mechanical ventilation, and both died. One patient with a confirmed case (A2.3) experienced mild symptoms of cough and subjective low-grade fever, as did the four others who received diagnoses of probable COVID-19. Two attendees did not develop symptoms within 14 days of the birthday party.

**FIGURE 2:**
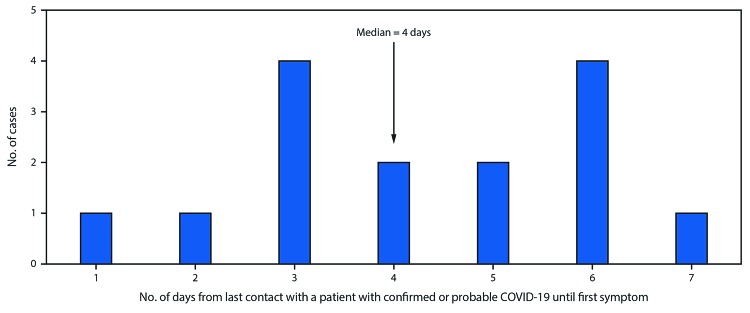
Likely incubation periods for confirmed and probable cases of COVID-19 following transmission of SARS-CoV-2 at two family gatherings (N = 15)* — Chicago, Illinois, February–March 2020 *The exposure of infection for the index patient, and consequently the incubation period, was unknown.

Two persons who provided personal care for patient A2.1 without using PPE, including one family member (patient A3.1) and a home care professional (patient C3.1), both developed probable COVID-19. It is likely that patient A3.1 subsequently transmitted SARS-CoV-2 to a household contact (patient A4.1), who did not attend the birthday party, but developed a new onset cough 3 days following unprotected, close contact with patient A3.1 while patient A3.1 was symptomatic.

Three symptomatic birthday party attendees with probable COVID-19 (patients A2.5, A2.6, and A2.7) attended church 6 days after developing their first symptoms (investigation day 17). Another church attendee (patient D3.1, a health care professional) developed confirmed COVID-19 following close contact with patients A2.5, A2.6, and A2.7, including direct conversations, sitting within one row for 90 minutes, and passing the offering plate.

The patients described in this report ranged in age from 5 to 86 years. The three patients who died (patients A2.1, B2.1 and A2.2) were aged >60 years, and all had at least one underlying cardiovascular or respiratory medical condition.

## Discussion

This cluster comprised 16 cases of COVID-19 (seven confirmed and nine probable), with transmission mostly occurring between nonhousehold contacts at family gatherings. The median interval from last contact with a patient with confirmed or probable COVID-19 to first symptom onset was 4 days. Within 3 weeks after mild respiratory symptoms were noted in the index patient, 15 other persons were likely infected with SARS-CoV-2, including three who died. Patient A1.1, the index patient, was apparently able to transmit infection to 10 other persons, despite having no household contacts and experiencing only mild symptoms for which medical care was not sought (patient A1.1 was only tested later as part of this epidemiologic investigation). Super-spreading events have played a significant role in transmission of other recently emerged coronaviruses such as SARS-CoV and MERS-CoV (*4*,*5*), although their relevance to SARS-CoV-2 spread is debated (*6*).

These data illustrate the importance of social distancing for preventing SARS-CoV-2 transmission, even within families. In this cluster, extended family gatherings (a birthday party, funeral, and church attendance), all of which occurred before major social distancing policies were implemented, might have facilitated transmission of SARS-CoV-2 beyond household contacts into the broader community. These findings support CDC recommendations to avoid gatherings (*7*) and reinforce the executive order from the governor of Illinois prohibiting all public and private gatherings of any number of persons occurring outside a single household (*8*).

The findings in this investigation are subject to at least three limitations. First, lack of laboratory testing for probable cases means some probable COVID-19 patients might have instead experienced unrelated illnesses, although influenza-like illness was declining in Chicago at the time. Second, phylogenetic data, which could confirm presumed epidemiologic linkages, were unavailable. For example, patient B3.1 experienced exposure to two patients with confirmed COVID-19 in this cluster, and the causative exposure was presumed based on expected incubation periods. Patient D3.1 was a health care professional, and, despite not seeing any patients with known COVID-19, might have acquired SARS-CoV-2 during clinical practice rather than through contact with members of this cluster. Similarly, other members of the cluster might have experienced community exposures to SARS-CoV-2, although these transmission events occurred before widespread community transmission of SARS-CoV-2 in Chicago. Finally, despite intensive epidemiologic investigation, not every confirmed or probable case related to this cluster might have been detected. Persons who did not display symptoms were not evaluated for COVID-19, which, given increasing evidence of substantial asymptomatic infection (*9*), means the size of this cluster might be underestimated.

In this cluster, two family gatherings outside the household likely facilitated the spread of SARS-CoV-2; one index patient who attended both events likely triggered a chain of transmission that included 15 other confirmed and probable cases of COVID-19 and ultimately resulted in three deaths. Media reports suggest the chain of transmission described in Chicago is not unique within the United States.^§^ Together with evidence emerging from around the world (*10*), these data shed light on transmission beyond household contacts, including the potential for super-spreading events. More comprehensive information is needed to better understand the transmission of SARS-CoV-2 in community settings and households to better inform initiation and termination of public health policies related to social distancing or stay-at-home orders. Overall, these findings highlight the importance of adhering to current social distancing recommendations,^¶^ including guidance to avoid any gatherings with persons from multiple households and following state or local stay-at-home orders.

**Summary**

**What is already known about this topic?**

Early reports of person-to-person transmission of SARS-CoV-2 have been among household contacts, health care workers, and within congregate living facilities.

**What is added by this report?**

Investigation of COVID-19 cases in Chicago identified a cluster of 16 confirmed or probable cases, including three deaths, likely resulting from one introduction. Extended family gatherings including a funeral and a birthday party likely facilitated transmission of SARS-CoV-2 in this cluster.

**What are the implications for public health practice?**

U.S. residents should adhere to CDC recommendations for social distancing, avoid gatherings, and follow stay-at-home orders when required by state or local authorities.

## Footnotes

* CDC. Evaluating and testing persons for coronavirus disease 2019 (COVID-19). https://www.cdc.gov/coronavirus/2019-ncov/hcp/clinical-criteria.html.

† For example, the likely index patient was the first person in the first generation and belonged to family A and is designated A1.1.

§ New York Times. After a funeral in a Georgia town, coronavirus ‘hit like a bomb.’ March 20, 2020. https://www.nytimes.com/2020/03/30/us/coronavirus-funeral-albany-georgia.html

¶ CDC. Coronavirus disease 2019 (COVID-19). How to protect yourself and others. Atlanta, GA: U.S. Department of Health and Human Services, CDC; 2020. https://www.cdc.gov/coronavirus/2019-ncov/prevent-getting-sick/prevention.html
